# Preventative Effects of *Cordyceps cicadae* Mycelial Extracts on the Early-Stage Development of Cataracts in UVB-Induced Mice Cataract Model

**DOI:** 10.3390/nu15143103

**Published:** 2023-07-11

**Authors:** Tsung-Han Lu, Jun-Way Chang, Bo-Yi Jhou, Jui-Hsia Hsu, Tsung-Ju Li, Li-Ya Lee, Yen-Lien Chen, Han-Hsin Chang, Chin-Chu Chen, Pey-Shiuan Wu, David Pei-Cheng Lin

**Affiliations:** 1 Department of Medical Laboratory and Biotechnology, Chug Shan Medical University, Taichung City 402, Taiwan; rightlu1114@gmail.com; 2 The Ph.D. Program of Biotechnology and Biomedical Industry, China Medical University, Taichung City 404, Taiwan; u103003404@cmu.edu.tw; 3 Grape King Bio Ltd., Taoyuan City 320, Taiwan; boyi.jhou@grapeking.com.tw (B.-Y.J.); juihsia.hsu@grapeking.com.tw (J.-H.H.); tsungju.li@grapeking.com.tw (T.-J.L.); ly.lee@grapeking.com.tw (L.-Y.L.); lan.chen@grapeking.com.tw (Y.-L.C.); gkbioeng@grapeking.com.tw (C.-C.C.); 4 Department of Ophthalmology, Chung Shan Medical University Hospital, Taichung City 402, Taiwan; jhhc@csmu.edu.tw; 5 Department of Nutrition, Chung Shan Medical University, Taichung City 402, Taiwan; 6 Department of Food Science, Nutrition, and Nutraceutical Biotechnology, Shih Chien University, Taipei City 104, Taiwan; 7 Institute of Food Science and Technology, National Taiwan University, Taipei City 106, Taiwan; 8 Department of Bioscience Technology, Chung Yuan Christian University, Taoyuan City 320, Taiwan; 9 Department of Cosmetic Science, Providence University, Taichung City 433, Taiwan

**Keywords:** cataract, ultraviolet B, TCM, *Cordyceps cicadae*, HSP

## Abstract

Cataracts, a prevalent age-related eye condition, pose a significant global health concern, with rising rates due to an aging population and increased digital device usage. In Taiwan, cataract prevalence is particularly high, reaching up to 90% among individuals aged 70 and above. The lens of the eye absorbs short-wave light, which can lead to oxidative stress in lens epithelial cells and contribute to cataract formation. Exposure to ultraviolet (UV) light further exacerbates the risk of cataracts by generating reactive oxygen species. Heat-shock proteins (HSPs), involved in protein maintenance and repair, have been linked to cataract development. Cordyceps cicadae (*C. cicadae*), a traditional Chinese medicine, has a long history of use and is known for its pharmacological effects. N6-(2-hydroxyethyl) adenosine (HEA), a bioactive compound found in *C. cicadae*, exhibits anti-inflammatory, immunomodulatory, and neuroprotective properties. Previous studies have shown that *C. cicadae* mycelial extracts improve dry eye disease and reduce intraocular pressure in animal models. Additionally, *C. cicadae* possesses antioxidant properties, which are beneficial for combating cataract formation. In this study, we aim to evaluate the preventive efficacy of *C. cicadae* mycelial extracts in UV-induced cataract development. By investigating the ameliorative effects of *C. cicadae* on eye diseases and its potential role in ocular health improvement, we hope to uncover new options for cataract prevention and provide insights into the mechanisms of action. The findings of this research could provide a novel approach for nutritional supplements targeting cataract prevention, offering potential benefits in the field of ocular health.

## 1. Introduction

Cataracts are a major cause of blindness and visual impairment strongly associated with aging [1], a problem that will only become more concerning due to the aging world population, contributing to heavier burden on caretakers and healthcare spending [2,3]. Currently, choices of effective treatment are largely limited to surgery and subsequent implantation of artificial lenses [4]. According to statistics in the East Asian country of Taiwan, the prevalence of cataract is 60% in people aged 50 and above, and up to 90% in people aged 70 and above.

With the advent of the digital age and the prevalence of digital devices, the everyday strain on the human eye has increased so much that names, such as “digital eye syndrome” and “computer vision syndrome”, have been coined to encompass the various symptoms associated with digital device usage [5]. The lens of the human eye absorbs short-wave light (400–490 nm), coincidentally the peak emission of the screens of smartphones and laptops, to protect the retina from light damage [6]. However, this action comes at the cost of increase in oxidative stress in human lens epithelial cells (hLECs), which has been a major suspect of cataract formation [7]. Combined with the already prevalent and serious hazard of solar ultraviolet (UV) radiation damage to the lens, risk factors of cataract have become omnipresent in our lives. Exposure to UV light or ionizing radiation, whether acute or prolonged, can lead to the generation of reactive oxygen species (ROS) as a byproduct of cellular energy metabolism. This process is recognized as a significant contributing factor in the development of cataracts [8]. A recent study has found that cataract patients have increased DNA damage in their lens epithelial cells [9]. This suggests a potential link between these damages caused by genotoxic stress and molecular chaperones of heat-shock proteins (HSPs), as they play a critical role in the adaptive response. HSPs protect intracellular proteins from misfolding or aggregation, which helps repair DNA damage and maintain cell integrity [10].

HSPs are a family of proteins that are produced in response to stress, including oxidative stress [11]. Evidence about the correlation between HSPs and cataracts have been reported [12,13]. Studies have shown that the expression of certain HSPs is altered in cataractous lenses compared to normal lenses [14]. HSPs are also produced in response to exposure to ultraviolet light and as a result of aging [15]. HSPs serve as molecular chaperones, assisting in the maintenance of the proper folding and function of various proteins in the cell. They can also prevent protein aggregation and degradation, further supporting damaged protein repairment.

*Cordyceps cicadae* (*C. cicadae*) are parasitic fungi of the cicada nymph or the larva of several species of cicadas [16]. The host body is consumed by the mycelium, which eventually sprouts flower-shaped stroma, earning its traditional name, Chanhua, or cicadae flower [17]. *C. cicadae* are a prized material in traditional Chinese medicine (TCM), having been consumed for around 1600 years. Many of the medicinal effects attributed to *C. cicadae* in ancient and imperial Chinese compendiums have been followed up on in modern times, as pharmacological research on *C. cicadae* have revealed a wide spectrum of actions and potential benefits including renal protective, anti-tumor, and immunomodulatory functions [18,19,20]. According to the present study, the positive effects observed with *C. cicadae* mycelium may be attributed to a specific bioactive component called N6-(2-hydroxyethyl) adenosine (HEA) [21,22]. HEA has been previously reported to possess several beneficial properties, including antioxidant, anti-inflammatory, immunomodulatory, and neuroprotective effects [23,24,25,26,27]. By exerting these effects, HEA may help mitigate the defective conditions associated with eye disease and contribute to an overall improvement in ocular health.

In a previous study, we explored the ameliorative effects of *C. cicadae* mycelial extracts on dry eye disease using a benzalkonium chloride (BAC)-induced mouse model [28]. Furthermore, we discovered that *C. cicadae* exhibited an alleviating effect on dry eye symptoms in humans [29]. Additionally, a study revealed that *C. cicadae* had an impact on reducing intraocular pressure in rats and rabbits [30]. Our previous research into eye health has shown promising results, motivating us to investigate other important aspects that have yet to be studied. Specifically, we discovered that HEA was present in brain tissue, indicating that it was able to penetrate the blood–brain barrier [31]. This suggests that the compound may have potential in alleviating eye diseases, thus, helping to reduce the effects of eye disease and contributing to overall improvements in ocular health. However, the connection between HEA-containing *C. cicadae* mycelial extracts and cataracts has not yet been researched. Therefore, this study aims to assess the effectiveness of *C. cicadae* mycelial extracts in preventing UV-induced cataract development in an animal model.

## 2. Materials and Methods

### 2.1. Sample Preparation

*C. cicadae* collected from the mountainous region in New Taipei City, Taiwan (24°48′57.0″ N 121°27′15.4″ E), underwent cultivation on potato dextrose agar for a period of 14 days at a temperature of 24 °C following strain isolation and authentication. After being identified, this specimen was preserved in herbaria at the Bioresource Collection and Research Center (BCRC) in Taiwan. HEA-enriched *C. cicadae* mycelia was obtained by introducing a mycelia agar block into a 1.0 L synthetic broth (comprising 1% soybean powder, 2% glucose, 1% yeast extract, with pH adjusted to 6.0 using 1N HCl) and cultivating it at 25 °C for 3 days on a rotary shaker set at 120 rpm. In order to achieve large-scale production, the fermentation process was scaled up from a 2 L shake flask to a 500 L fermenter, employing the same growth conditions. The fermented HEA-enriched *C. cicadae* mycelia were subsequently subjected to boiling at 100 °C for 3 h, followed by lyophilization and grinding into a powder, and stored at 4 °C. The freeze-dried powder was added to water and ethanol separately to extract the contents. The *C. cicadae* mycelia were extracted with hot water (100 °C) and subsequently filtered. The filtrate was collected and dried through lyophilization. Additionally, the mycelia were extracted twice with 95% ethanol over a seven-day period, and dried using rotary evaporation. The two different extracts were then resuspended at 100 ppm using either 0.9% NaCl aqueous solution or commercial soybean oil (SBO) (Taisugar) to be fed to mice. A chemical analysis was conducted on *C. cicadae* mycelia (*C. cicadae* mycelial water extracts and *C. cicadae* mycelial ETOH extracts), which included a quantification of the content of HEA (C12H17N5O5). HEA was quantified according to a previous study [32]. Briefly, *C. cicadae* mycelial water extracts and *C. cicadae* mycelial ETOH extracts were redissolved in 20 volumes of solvent, and the supernatant was centrifuged by ultrasonic wave and filtered through a 0.45 pm filter. High-performance liquid chromatography (HPLC) (Hitachi L-5000 Series) with a UV wavelength 254 nm was carried out using a reverse-phase separation column (Inertsil@, ODS-2, 4.6 × 250 mm, 5 um). This method involved the use of a binary gradient containing a mobile phase consisting of (A) deionized water and (B) acetonitrile. The solvent gradient elution procedure was as follows: gradient 0–15 min 100% A, 15–40 min 100–80% A, 40–55 min 80–50% A, 55–75 min 50–0% A, 75–90 min 0% A, 90–91 min 0–100% A, and 91–100 min 100% A. The flow rate was 1.0 mL/min, and 10 µL was injected, including the standards and samples.

### 2.2. Animals and Feeding

**Figure 1:**
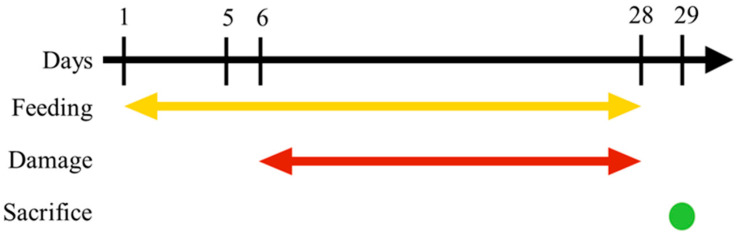
The timeline of this experiment. Mice were given treatments starting on day 1, and UVB damage was administered daily between 9 and 10 a.m. from days 6 to 28. On day 29, the mice were sacrificed, and their eye tissue was collected for evaluation.

Six-week-old female ICR mice were purchased from BioLASCO Taiwan Co., Ltd., Taipei, Taiwan, and were kept in animal room maintained at 25 ± 1 °C, 55 ± 5% humidity, with 12 fresh-air changes per hour. Mice were divided into 6 groups of 3 per group (*n* = 3), with 18 mice in total. The naming, UVB treatment, and test samples administered to each of the 6 groups are described in Table 1. Test samples were administered by oral gavage once per day between 8 and 9 a.m. with total volume of 0.2 mL. The timeline of this experiment is shown in Figure 1. Briefly, test samples were administered starting from day 1 of the experiment, while UVB damage was administered each day between 9 and 10 a.m. on days 6 through 28. The mice were sacrificed on day 29 and their eye tissue was taken for assessment. All experiment protocols were approved by the Institutional Animal Care and Use Committee of Chung Shan Medical University, Taichung, Taiwan (Approval number: 1516) and were compliant with the Association for Research in Vision and Ophthalmology (ARVO) Resolution on the Use of Animals in Research.

### 2.3. UVB Damage Induction

Mice pupils were dilated with 1% tropicamide one minute prior to being anaesthetized. Once mice entered anesthesia, researchers manually blinked the mice’s eyes several times to preserve integrity of the tear film. Afterwards, mice were placed in a light box (CN-6 darkroom, Vilber Lourmat, Germany) with an attached UV lamp (Uvitec LF-206LS, Cambridge, UK; tube: Vilber-Lourmat T-6M). Each eye was damaged with UVB for 50 s.

### 2.4. Lens and Eyeball Imaging Test

Mice were sacrificed by carbon dioxide asphyxiation, after which the right eye was removed and placed on a white background with a horizontal red line such that the corneal limbus was aligned with the red line. Photographs of the side view of the eye were taken such that the red line can be visualized through the side of the lens. The left eye was taken, and the lens was removed and placed on a grid or dotted background. Top-down photos were taken.

### 2.5. Immunohistochemistry (IHC) Stainning

The paraffin-embedded cortex and lens epithelial tissues from mice eyes were deparaffinized and rehydrated. The slides were stained with HSP27 (1:200, ab109376), HSP47 (1:250, ab109117), HSP70 (1:300, ab181606), and HSP90 (1:200, ab109248) antibody, biotin-conjugated goat anti-rabbit immunoglobulin G (IgG) was used as the secondary antibody, and 3,3′-diaminobenzidine tetrahydrochloride was used as the substrate for color development. Finally, the slides were mounted and photographed under a light microscope (Nikon SMZ645, Tokyo, Japan) for examination of the changes in oxidative stress.

### 2.6. Terminal Deoxynucleotidyl Transferase dUTP Nick End Labeling (TUNEL) Assay

Paraffin-embedded lens epithelial tissue sections were deparaffinized and rehydrated. Slides were then incubated in phosphate-buffered saline with Tween-20 (PBST) solution for 10 min for the purpose of permeabilization. After washing with PBS, 50 μL of TUNEL reaction mixture (Roche Applied Science–12156792910, Basel, Switzerland) was added to each epithelial section, and sections were then incubated at 37 °C for 1 h in a humidified chamber. After several washes, slides were mounted, and fluorescence images were taken with a fluorescence microscope (Zeiss, Jena, Germany) at 100× and 400×.

## 3. Results

### 3.1. Proximate Composition Analysis of HEA in C. cicadar Mycelial Water and Ethanol Extracts

**Figure 2:**
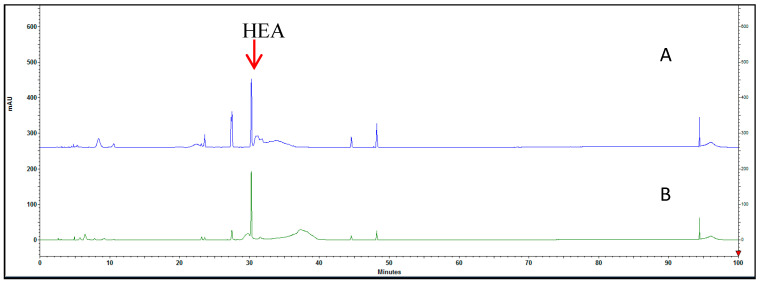
High-performance liquid chromatogram of HEA-enriched *C. cicadae* mycelial extracts (**A**) HPLC chromatogram of the *C. cicadae* mycelial water extract. (**B**) HPLC chromatogram of the *C. cicadae* mycelial water extract.

HEA is the primary pharmacologically active component found in *C. cicadae* [33]. However, the role of HEA-enriched *C. cicadae* in relation to cataracts remains unclear. Hence, an analysis of their concentration in the mycelium extract was conducted using HPLC. The retention time for HEA was determined to be 30.15 min, and the content of HEA in the water extract of *C. cicadae* mycelium (3.9 mg/g) was found to be higher than that in the ethanol extract of *C. cicadae* mycelium (2.9 mg/g) (Figure 2A,B).

### 3.2. C. cicadae Ameliorate the Severity of Lens Opacity in Cataract

**Figure 3:**
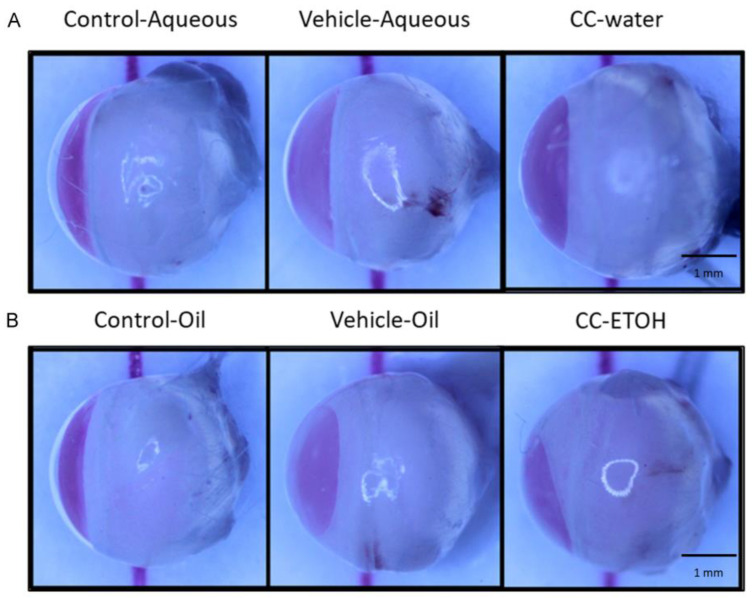
Slit lamp analysis of ICR mice. (**A**) Representative images of crystal lens from aqueous group. (**B**) Representative images of crystal lens from oil group. Scale bar = 1 mm. CC-water: *C. cicadae* mycelial water extract; CC-ETOH: *C. cicadae* mycelial ethanol extract.

In this experiment, the side view of the lenses was examined. The degree of lens opacity and transparency was directly observed by comparing the shade of red in each group. No abnormalities were observed in the lenses of undamaged control groups (Control-Aqueous and Control-Oil) (Figure 3A,B) as the red color was most intense, whereas significant opacity was seen in the lenses of damaged groups treated solely with solvent (Vehicle-Aqueous and Vehicle-Oil) which showed a lighter shade of red. Thus, the UVB induction of cataract symptoms was confirmed. In samples of *C. cicadae* mycelial water extract and *C. cicadae* mycelial ethanol extract, the red color was markedly more intense than in the damage groups and comparable to that of the blank groups, indicating a lesser degree of lens opacity progression and better transparency.

### 3.3. C. cicadae Decelerate the Development of Cataract

**Figure 4:**
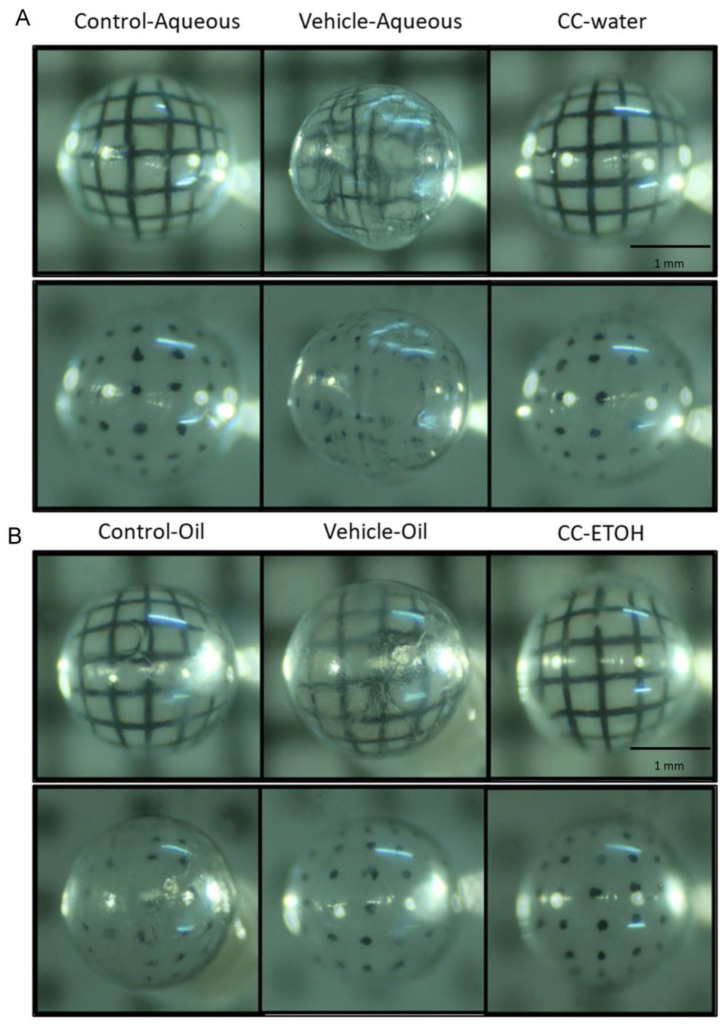
Crystal lens images of ICR mice. (**A**) Representative images of crystal lenses from aqueous group. (**B**) Representative images of crystal lenses from oil group. Scale bar = 1 mm. CC-water: *C. cicadae* mycelial water extract; CC-ETOH: *C. cicadae* mycelial ethanol extract.

The opacity and image distortion of the lens was further observed using a grid background and a dotted background. Severe distortion of the background and mild opacity was observed in the lens of vehicle groups when compared to those of undamaged control group. The lines of the grid appeared to be interrupted and twisted (Figure 4A,B) while the dots became displaced and blurred. The images of the groups with *C. cicadae* mycelial water extract and *C. cicadae* mycelial ethanol extract were discernable from those of the control group, indicating that these two samples provided protection against cataract development.

### 3.4. C. cicadae Mycelium Suppres HSPs in the Cortex of Crystalline Lens

**Figure 5:**
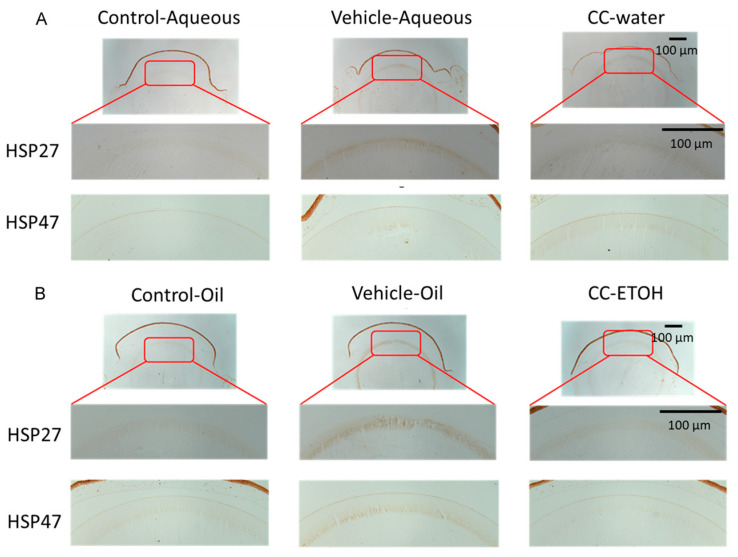
*C. cicadae* suppresses HSP27 and HSP47 expression in the cortex. (**A**) Representative images of Hsp 27 and Hsp 47 staining from aqueous group. (**B**) Representative images of Hsp 27 and Hsp 47 staining from oil group. Scale bar = 100 µm. CC-water: *C. cicadae* mycelial water extract; CC-ETOH: *C. cicadae* mycelial ethanol extract; HSP27: heat-shock protein 27; HSP47: heat-shock protein 47.

The expression of HSPs is induced in response to oxidative stress, and plays a key role in protecting cells from the damaging effects of ROS in eyes [34]. The cortex of the lens in the UVB-damaged aqueous and oil groups expresses a large amount of HSP27 and HSP47 compared to the cortex in control group (Figure 5A,B). In both treatment groups, the expression of HSP27 and HSP47 decreased after feeding with *C. cicadae* mycelial water extract and ethanol extract compared with UVB-damaged groups.

### 3.5. C. cicadae Mycelium Suppress HSPs in hLECs

**Figure 6:**
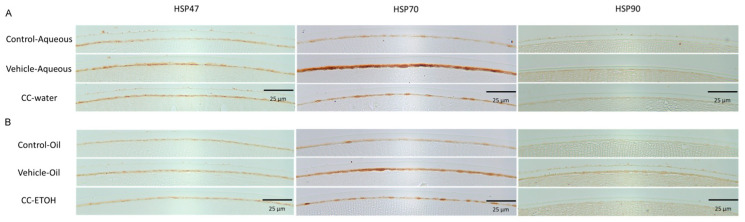
*C. cicadae* decreases HSP47, HSP70, and HSP90 expression in hLECs. (**A**) Representative images of Hsp 47, Hsp 70, and Hsp 90 staining from aqueous group. (**B**) Representative images of Hsp 47, Hsp 70, and Hsp 90 staining from oil group. Scale bar = 25 μm. CC-water: *C. cicadae* mycelial water extract; CC-ETOH: *C. cicadae* mycelial ethanol extract; HSP47: heat-shock protein 47; HSP70: heat-shock protein 70; HSP90: heat-shock protein 90.

In the UVB-damaged aqueous group, the levels of HSP47, HSP70, and HSP90 expression were markedly increased compared with control group, and similar results were found in the UVB-damaged oil group (Figure 6A,B). These values were significantly decreased by *C. cicadae* mycelial extracts when compared with UVB-damaged (Figure 6A,B).

### 3.6. C. cicadae Mycelium Suppres Apoptosis of hLECs

**Figure 7:**
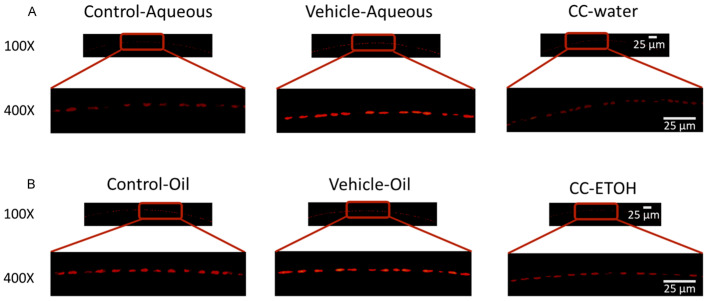
*C. cicadae* ameliorates the apoptotic rates in hLECs. (**A**) Representative images of TUNEL staining from aqueous group. (**B**) Representative images of TUNEL staining from oil group. Scale bar = 25 μm. CC-water: *C. cicadae* mycelial water extract; CC-ETOH: *C. cicadae* mycelial ethanol extract.

Apoptosis of hLECs is one of the mechanisms that can lead to cataract formation. We used TUNEL staining to examine the apoptotic status of the nuclei in lens epithelial cells. The apoptotic status of the cells in both damage groups were severe, but after taking *C. cicadae* mycelium, the number of apoptotic nuclei sharply decreased (Figure 7A,B), demonstrating that *C. cicadae* mycelial extract has a protective effect in preventing apoptosis of lens epithelial cells.

## 4. Discussion

Cataracts affect numerous people in the world; although the natural aging process of the eye is a main reason, oxidative stress is also thought to play a role in the development of cataracts [35,36]. UVB-induced oxidative damage is a critical triggering factor for cataracts [37]. The lens in the eye has its own built-in mechanisms to protect against the harmful effects of UVB and ROS. However, if the amount of oxidative stress caused by UVB exposure is too much for the lens to sustain, taking external antioxidant supplements becomes crucial for preventing the formation of cataracts. Therefore, finding a supplement with antioxidant properties is a vital goal in slowing down the development of cataracts. *C. cicadae* has been reported to contain antioxidant and anti-inflammatory effects in several previous study [30,32,38,39]. Moreover, a clinical study found *C. cicadae* can ameliorate dry eye symptoms [29], demonstrating that the potential of using *C. cicadae* in eye diseases. Here, we found that *C. cicadae* mycelium extracts exhibit a preventative effect on UVB-induced cataracts. This investigation suggests a new role of *C. cicadae* in application of eye disease.

Currently, there are no drugs available in Western medicine to treat cataracts, which begin with the opacification of the lens. Traditional Chinese medicine offers several treatments for cataracts, including acupuncture, herbal medicine, and dietary therapy, as described in ancient TCM texts [40]. However, the efficacy of TCM in treating cataracts is currently limited by modern research. A recent study revealed a protective effect of *Lycium barbarum*, a wildly used Chinese herb, against diabetic cataracts [41]. Moreover, *D. huoshanense*, a traditional Chinese medicine, has been found to possess anticataract activity by inhibiting oxidative stress and apoptosis in human lens epithelial cells, potentially offering a preventive and curative approach for diabetic cataracts [42]. In our study, *C. cicadae* extracts show the ability to ameliorate severe opacity and transparency of cataract in mice. Thus, we have successfully demonstrated the function of *C. cicadae* mycelium extracts to stall cataract development in its early stages, or, in other words to retard the opacification of the lens, demonstrating a novel insight of using TCM in cataract therapy.

The positive effects observed in the current study can be attributed to a specific bioactive compound found in *C. cicadae* mycelium called HEA. Previous reports have highlighted the anti-inflammatory, immunomodulatory, and neuroprotective properties of HEA [23,24,25,26]. In our research, we observed that the water extract of *C. cicadae* exhibits a superior preventive effect compared to the ethanol extract. This observation may be attributed to the variation in the amount of HEA present in each extract. Specifically, the content of HEA in the *C. cicadae* water extract is 3.9 mg/g, slightly higher than that in the ethanol extract. Furthermore, when utilizing the ethanol extraction method, there is a possibility that impurities in the substance are more likely to dissolve, leading to distinct outcomes when compared to the water extraction group.

The ocular tissues contain adenosine receptors (ARs), and recent studies have identified A1AR, A3AR, and A2AAR agonists as inflammation modulators. Adenosine, along with its analog HEA, is the primary physiologically active component of *C. cicadae* and may potentially act through ARs to modulate oxidative stress on the ocular surface. While adenosine does play a role in modulating inflammation and oxidative stress in certain contexts, its direct involvement in cataract formation is still uncertain. Some studies suggest the involvement of adenosine or its receptor in cataract development [43,44]; however, research into the mechanisms underlying cataract formation is still ongoing. Further study is needed in the future for new insights.

HSPs play vital roles in cellular processes, including the regulation of intercellular reactive oxygen species (ROS), maintenance of glutathione levels, control of cell apoptosis, and preservation of cell structure. Previous research has demonstrated that HSP27 exhibits an increase in expression and protects the lens when subjected to external stress [45]. Additionally, elevated expression of HSP47 is closely associated with the excessive deposition of collagen, suggesting its involvement in fibrotic diseases and processes characterized by increased collagen expression [46]. In our findings, HSP27 and HSP47 increased during UVB-damage, but decreased after receiving *C. cicadae* mycelium, indicating that *C. cicadae* can reduce the damage caused by oxidative stress after exposure to UV radiation, alleviate massive deposition of collagen, and maintain the cortex from further fibrosis.

In the presence of oxidative stress, the expression of HSP70 is upregulated to facilitate cellular recovery from oxidative damage and provide protection against subsequent harm [47]. Another study conducted by Zhang et al. investigated the heat-shock response in human lens epithelial cells (hLECs) and confirmed that HSP90 levels increase in response to cellular stress or damage, thereby safeguarding lens proteins [48]. Furthermore, this response was found to be time-dependent, suggesting a regulated mechanism. In this study, HSP70 and HSP90 expression is sharply increased in UVB-damaged aqueous and oil groups, while after intervention with *C. cicadae* mycelium, the expression of HSP70 decreased significantly, demonstrating that *C. cicadae* mycelium can help lens epithelial cells recover from oxidative stress and assist in correct protein folding.

Extracts from *C. cicadae* mycelium have demonstrated significant potential as a dietary supplement for the prevention and protection against cataracts. Further research investigating the combination of eyedrops and dietary supplements containing *C. cicadae* mycelium, as well as the possibility of solely relying on the supplement for delaying cataracts, presents an intriguing opportunity for cataract prevention. Currently, the primary approach for treating cataracts involves a surgical procedure, which can be costly and necessitates the removal of the cataract lens. While this surgery is covered by health insurance in Taiwan, many countries worldwide do not provide this benefit. Consequently, this supplement holds particular value for populations lacking access to surgical services, including those in developing countries or low-income communities. This study not only sheds light on the potential advantages for cataract patients but also introduces a novel option for cataract prevention to the global community.

## 5. Conclusions

In summary, this is the first study to investigate the functions of *C. cicadae* in cataract prevention. The eye and lens characteristics of mice fed with *C. cicadae* mycelial water extract and *C. cicadae* mycelial ethanol extract prior to UVB damage are comparable to those of mice that have not undergone damage. Furthermore, mice fed with *C. cicadae* mycelial water extract and *C. cicadae* mycelial ethanol extract showed that the extracts abolished the oxidative stress induced by UVB damage. Finally, TUNEL staining revealed the protective effect of *C. cicadae* in UVB damage-induced cell apoptosis. These findings indicate that *C. cicadae* mycelium can counteract oxidative damage in mice with cataract, suggesting dietary supplementation with *C. cicadae* may serve as a promising strategy for preventing or mitigating cataract formation.

## Figures and Tables

**Table 1:** Animal grouping and treatment.

Name	Name	Feed	UVB Damage
Aqueous	Control-Aqueous	0.9% NaCl solution	No damage
	Vehicle-Aqueous	0.9% NaCl solution	Damage, days 6–28
	*C. cicadae* mycelial water extract	Extract in 0.9% NaCl solution	Damage, days 6–28
Oil	Control-Oil	SBO	No damage
	Vehicle-Oil	SBO	Damage, days 6–28
	*C. cicadae* mycelial ethanol extract	Extract in SBO	Damage, days 6–28

## Data Availability

The raw materials for this study are available from the corresponding authors.
